# Editorial: Community and clinical pharmacy services in type 2 diabetes care, volume II

**DOI:** 10.3389/fcdhc.2026.1812553

**Published:** 2026-03-11

**Authors:** Muhammad Shahzad Aslam

**Affiliations:** 1 Western Medicine Unit, School of Traditional Chinese Medicine, Xiamen University Malaysia, Sepang, Malaysia; 2 Department of Traditional Chinese Medicine, School of Medicine, Xiamen University, Xiamen, Fujian, China

**Keywords:** diabetes mellitus, diabetes - quality of life, health profession, medication, pharmacist and nurse

**Figure 1:**
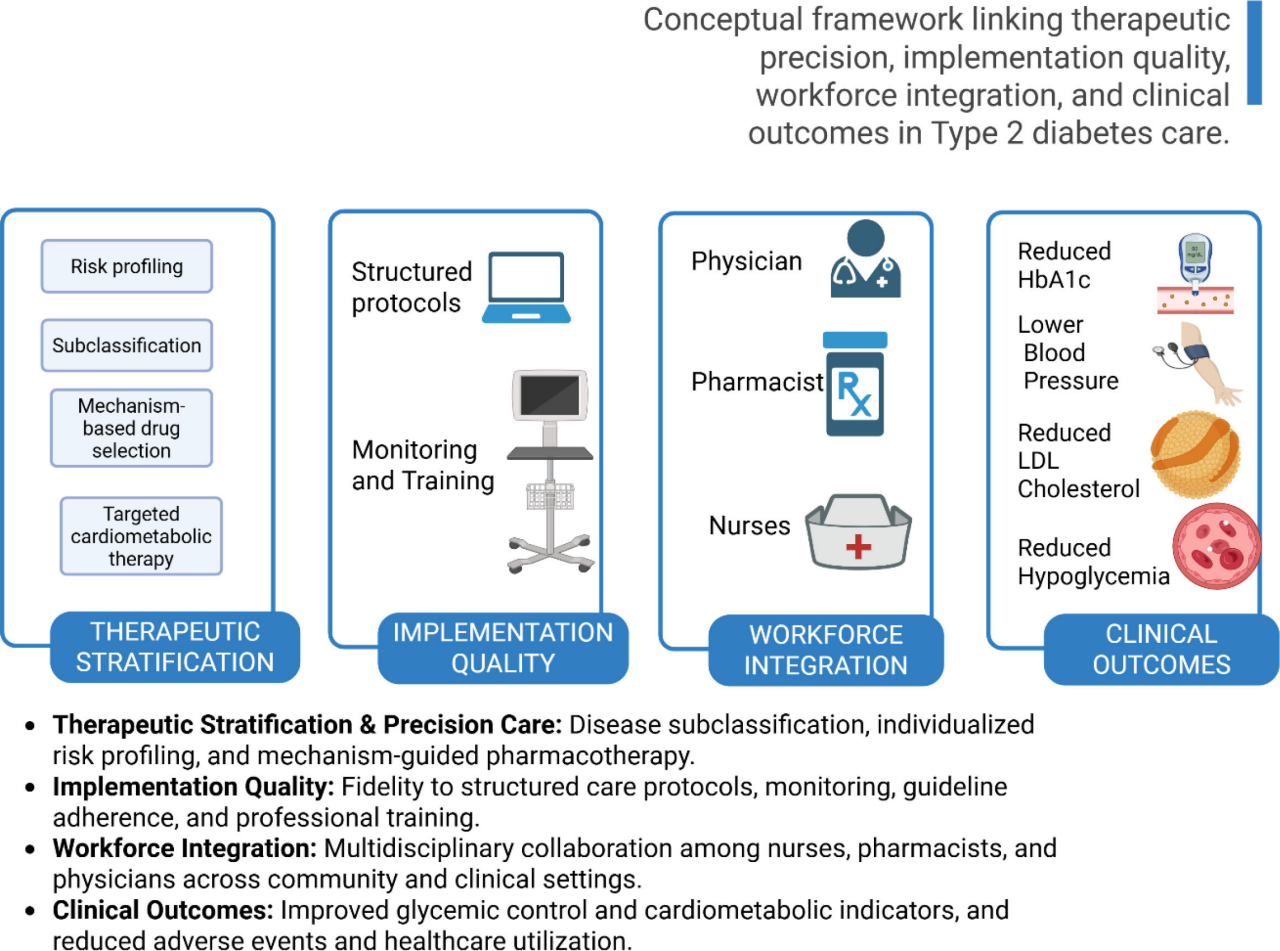
Conceptual framework linking therapeutic precision, implementation quality, workforce integration, and clinical outcomes in Type 2 diabetes care.

Diabetes mellitus is a leading noncommunicable disease, with global cases rising from 108 million in 1980 to 476 million in 2017. Prevalence among adults doubled to 8.5% by 2014. Diabetes causes 1.4 million deaths annually, driving significant health, social, and economic burdens worldwide (1). Its management extends beyond glycemic control to include cardiovascular risk reduction, complication prevention, and sustained patient engagement. Type 2 diabetes and cardiovascular disease are interconnected conditions within a cardiometabolic continuum, driven by shared biological mechanisms. Newer therapies like SGLT2 inhibitors, GLP-1 receptor agonists, and tirzepatide improve cardiorenal outcomes, but disparities in access and response remain. Integrated, equitable care models are vital for effective, personalized treatment (2). **Figure 1** illustrates a conceptual framework linking therapeutic precision, implementation quality, workforce integration, and clinical outcomes in Type 2 diabetes care. Pharmacists and nurses play increasingly critical roles in multidisciplinary care, particularly in community and transitional settings where continuity and adherence remain major challenges. This Research Topic, Community and clinical pharmacy services in type 2 diabetes care: Volume II, brings together important contributions that examine the clinical effectiveness, implementation quality, and professional capacity underlying integrated diabetes care models. Implementation fidelity represents a key determinant of intervention effectiveness (3). In an implementation study by Sun et al., of 3,351 adults aged ≥65 years, fidelity varied (mean 0.64, SD 0.19; range 0.28–0.94), and higher fidelity was associated with lower HbA1c (adjusted β −0.38 per 0.10-unit increase, 95% CI −0.47 to −0.29; p < 0.001), with a graded quartile pattern [7.89% (95% CI 7.78–8.00) vs 7.16% (95% CI 7.04–7.28); p for trend < 0.001], alongside lower systolic blood pressure (−5.10 mmHg, 95% CI −7.20 to −3.00), lower LDL cholesterol (−6.50 mg/dL, 95% CI −9.10 to −3.90), reduced hospitalization (IRR 0.61, 95% CI 0.51–0.73; p < 0.001), and lower odds of hypoglycemic events (OR 0.78, 95% CI 0.72–0.84; p < 0.001).These findings highlight that structured nursing-led assessments can deliver meaningful clinical benefits when implemented consistently, emphasizing the importance of implementation quality and workforce preparedness.

Pharmacist-integrated care models represent another promising strategy for strengthening diabetes management (4). A study by Gao et al., titled Construction and effectiveness of a pharmacist-involved diabetes management model between tertiary hospitals and community under the hierarchical medical system, enrolled 210 patients and evaluated a pharmacist-involved collaborative care model over 12 months. HbA1c, the primary endpoint, decreased in the intervention group, with values declining from 7.70 ± 2.19% at baseline and reaching 6.50 ± 1.07% after 12 months, and follow-up HbA1c remained significantly lower than in the control group receiving routine doctor-led care [7.10 (6.40, 7.80); between-group comparison p < 0.001]. The pharmacist-integrated model significantly improved glycemic indicators and medication adherence, demonstrating the value of pharmacist participation in bridging care gaps across healthcare levels and improving chronic disease management outcomes. A study by Kathleen A Johnson et al., indicate that the integration of clinical pharmacy services in safety net medical homes improved diabetes outcomes. Patients had greater reductions in A1C and were three times more likely to achieve treatment goals than controls (5).

Professional competency among frontline providers is equally essential for effective diabetes care (6). The cross-sectional study by Lan et al., involving 1,911 community nurses, reported poor insulin injection knowledge in 47.7% of participants, while poor attitude and poor practice were uncommon (3.7% and 2.5%, respectively); familiarity with clinical guidelines and insulin injection training were significant predictors of knowledge, attitude, and practice (all p < 0.05). Training exposure and familiarity with clinical guidelines were significantly associated with improved knowledge, attitude, and practice (7). These findings underscore the importance of targeted professional education to ensure safe and effective insulin administration as diabetes care increasingly shifts toward community settings.

Beyond care delivery structures, advances in therapeutic stratification are also critical for improving outcomes (8). The review article, Reyes-Medina et al., highlights the heterogeneity of T2DM and notes that patients with recently diagnosed disease may be classified into seven pathophysiologically distinct subgroups, emphasizing that cardiovascular disease represents a major complication and that stratified classification may support more precise and individualized therapeutic approaches. By aligning pharmacological strategies with underlying disease mechanisms, subclassification offers a pathway toward more personalized and effective treatment.

The studies included in this Research Topic collectively identify several priorities for advancing diabetes care. Implementation fidelity is essential for achieving significant clinical improvements, underscoring the need for structured protocols and comprehensive workforce training. Pharmacist-integrated care models present scalable approaches to enhance patient outcomes and reinforce continuity of care within healthcare systems. Maintaining professional competency among nurses and pharmacists remains crucial for safe and effective diabetes management, especially in community-based settings. Furthermore, recent progress in subclassification and therapeutic stratification offers new possibilities for personalized care that addresses the heterogeneity of the disease.

With the global rise in diabetes prevalence, strengthening integrated pharmacy and nursing services is essential to enhance care quality and accessibility. The findings in this Research Topic indicate that advancements in care delivery, workforce capacity, and therapeutic precision yield measurable clinical benefits. Ongoing investment in multidisciplinary collaboration, professional education, and implementation science is necessary to achieve sustainable improvements in diabetes outcomes.
